# Commensal *Escherichia coli* Antimicrobial Resistance and Multidrug-Resistance Dynamics during Broiler Growing Period: Commercial vs. Improved Farm Conditions

**DOI:** 10.3390/ani11041005

**Published:** 2021-04-03

**Authors:** Laura Montoro-Dasi, Arantxa Villagra, Sandra Sevilla-Navarro, Maria Teresa Pérez-Gracia, Santiago Vega, Clara Marin

**Affiliations:** 1 Instituto de Ciencia y Tecnología Animal, Universidad Politécnica de Valencia, 46022 Valencia, Spain; laura.montoro@outlook.com; 2 Centro de Calidad Avícola y Alimentación Animal de la Comunidad Valenciana (CECAV), 12539 Castellón, Spain; s.sevilla@cecav.org; 3 Centro de Investigación y Tecnología Animal, Instituto Valenciano de Investigaciones Agrarias, 12400 Castellón, Spain; villagra_ara@gva.es; 4 Departamento de Producción y Sanidad Animal, Salud Pública Veterinaria y Ciencia y Tecnología de los Alimentos, Instituto de Ciencias Biomédicas, Facultad de Veterinaria, Universidad Cardenal Herrera-CEU, CEU Universities, Avenida Seminario s/n, 46113 Moncada, Spain; svega@uchceu.es; 5 Área de Microbiología, Departamento de Farmacia, Instituto de Ciencias Biomédicas, Facultad de Ciencias de la Salud, Universidad Cardenal Herrera-CEU, CEU Universities, Avenida Seminario s/n, 46113 Moncada, Spain; teresa@uchceu.es

**Keywords:** antimicrobial resistance, multidrug resistance, broiler, farm management, *Escherichia coli*

## Abstract

**Simple Summary:**

This experiment was designed to evaluate the differences in antimicrobial and multidrug resistance dynamics in broilers reared under two different farm conditions (commercial vs. improved) during the growing period, using *Escherichia coli* as sentinel bacterium. Although no antibiotics were applied during rearing for two different management conditions tested, high rates of antimicrobial and multidrug-resistant bacteria were observed throughout rearing, with the percentages of resistant bacteria observed being of particular concern in day-old chicks on arrival day and in chickens at the end of the growing period, just before delivery to the slaughterhouse.

**Abstract:**

New measures applied to reduce antimicrobial resistances (AMR) at field level in broiler production are focused on improving animals’ welfare and resilience. However, it is necessary to have better knowledge of AMR epidemiology. Thus, the aim of this study was to evaluate AMR and multidrug resistance (MDR) dynamics during the rearing of broilers under commercial (33 kg/m^2^ density and max. 20 ppm ammonia) and improved (17 kg/m^2^ density and max. 10 ppm ammonia) farm conditions. Day-old chicks were housed in two poultry houses (commercial vs. improved), and no antimicrobial agents were administered at any point. Animals were sampled at arrival day, mid-period and at slaughter day. High AMR rates were observed throughout rearing. No statistical differences were observed between groups. Moreover, both groups presented high MDR at slaughter day. These results could be explained by vertical or horizontal resistance acquisition. In conclusion, AMR and MDR are present throughout rearing. Moreover, although a lower level of MDR was observed at mid-period in animals reared under less intensive conditions, no differences were found at the end. In order to reduce the presence of AMR bacteria in poultry, further studies are needed to better understand AMR acquisition and prevalence in differing broiler growing conditions.

## 1. Introduction

Antimicrobial resistance (AMR) is one of the most significant threats to public health worldwide. Indeed, the World Health Organisation published that by 2050, if effective interventions against the increase in AMR are not carried out, there could be more than 10 million deaths annually as a result of such resistance [1]. Increased awareness of the health threats related to AMR has resulted in greater social demand for antibiotic-free food production, especially antibiotic-free meat, in recent years [2,3,4,5,6,7].

The European Medicines Agency reported that Spain has been the European country with the highest consumption of antimicrobial agents (AMAs) since data became available [8]. In this sense, it is claimed that the uncontrolled administration of AMAs in the past, as treatment for infectious diseases or as a growth promoter, has resulted in an increased multidrug resistance (MDR) presence in the food chain [9,10,11]. In fact, the notable prevalence of colistin resistance is particularly worrying, due to its widespread use in veterinary medicine over many years, as it is a last-resort AMA reserved to treat MDR bacterial infections in human medicine [12].

However, due to the strict control of AMA administration since the National AMR Plan was established in 2014, their consumption in animal production has halved [13]. Specifically, between 2015 and 2019, in poultry a reduction of 71% in total AMA administration was reported, along with a 95% falloff in colistin administration, recording the largest European drop in consumption of critical AMAs [14].

These data are the result of the efforts carried out by the poultry sector to reduce AMA administration at field level. Firstly, by avoiding the entry and spread of pathogen microorganisms, improving biosecurity, farm management and vaccination protocols [15]; and secondly, by investing in more accurate and animal-friendly management systems, achieving animals with a strengthened immune system and more resilient to contact with infectious agents [16,17,18,19]. To this end, the use of alternative production systems has been promoted, focused on slow-growing breeds selected for their ability to deal with the natural environment [20], and the implementation of less intensive production systems, more sustainable and animal-welfare-friendly, but also maintaining the profitability of broiler farms [21,22].

However, to be able to assess the effectiveness of these measures, it is necessary to have better knowledge of the epidemiology of AMR throughout the growing period under different farm conditions [23,24]. For this purpose, commensal *Escherichia coli* has typically been selected as AMR sentinel, as it provides valuable data and constitutes a reservoir of resistance genes, which can spread to zoonotic and other bacteria [20,25].

Nevertheless, further studies are still needed to achieve more resilient animals to ensure that AMA administration continues to decrease at field level. In this context, the aim of this study was to evaluate the AMR and MDR dynamics in broiler chickens during the rearing period under two different management conditions (commercial vs. improved), using *Escherichia coli* as sentinel bacterium.

## 2. Materials and Methods

In this experiment, animals were handled according to the principles of animal care published by Spanish Royal Decree 53/2013 [26]. Moreover, all protocols were approved by the Ethical Review Panel of the Directorate-General for Agriculture, Fisheries and Livestock from the Valencian Community, by the code 2018/VSC/PEA/0067.

### 2.1. Experimental Design

The study was carried out in an experimental poultry farm at the Centre for Research and Animal Technology (CITA-IVIA, in its Spanish acronym *Valencian Institute for Agrarian Research*, Segorbe, Spain). The cleaning and disinfection protocol applied in the poultry farm was according to the Kersia Group protocol [27]. The product used to clean the poultry houses was Hyprelva Net Plus (Hypred S.L., Orcoyen, Spain), and the product employed to disinfect them was Virobacter (Hypred S.L., Orcoyen, Spain). Finally, the product used to disinfect the pipelines was Deptal SMP 5% (Hypred S.L., Orcoyen, Spain).

A total of 1062 day-old chicks (Ross^®^) (males and females) were housed in two identical poultry houses (531 animals in each house). Within each of the houses, 204 animals were located in 12 pens and the rest of them (327) were on the floor out of the pens, all with wood shavings as bedding material. Moreover, two different management conditions were evaluated: commercial farm conditions (CFC, house 1) and improved farm conditions (IFC, house 2). In house 1 (CFC) animals were kept at 33 kg/m^2^ density and non-optimal parameters of ventilation were applied (allowing a maximum concentration of ammonia of 20 ppm), while in house 2 (IFC) chicks were kept at 17 kg/m^2^ density and ventilation was provided within the optimal parameters (allowing a maximum concentration of ammonia of 10 ppm). Ammonia concentration was continuously measured from the air, using a Exafan climatic sensor DOL 53, installed near the outlet to obtain representative values of the room concentrations. Moreover, both houses were equipped with programmable electrical lights, automated electric heating and forced ventilation. The lighting program was decreasing from 23L:1D on the arrival day to 16L:8D from day 15 to the end of the growing period. Light intensity was guaranteed to be at least 20 lux in all parts of the farm at the height of the animals, and the light was provided through white bulb lamps uniformly distributed throughout the poultry house. The environmental temperature was set at 32 °C on arrival day and gradually reduced to 19 °C by 41 days post hatching in line with common practice in poultry production.

Day-old chicks were vaccinated in the hatchery against Gumboro disease, Marek disease and infectious bronchitis (IBV). During the growing period, no vaccines were administered.

Animals received drinking water and were fed ad libitum; feed was weighed and distributed manually. Two different age commercial diets were used to meet animals’ metabolic requirements (Table 1): from arrival day until 21 days post hatch, a pelleted starter diet was offered to the birds (Camperbroiler iniciación, Alimentación Animal Nanta, Valencia, Spain), and from 21 days old until slaughter day they were fed a pelleted grower diet (A-32 broiler, Alimentación Animal Nanta, Valencia, Spain). Nutritional and product analysis were assessed before the arrival of animals and only one batch of feed per age was manufactured. Moreover, no coccidiostats or AMAs were added to either diet, and high biosecurity levels were maintained in the experimental poultry house during the rearing. Finally, the mortality rates and presence of diarrhea were registered daily, and animals’ weight and feed consumption were recorded at weekly intervals.

### 2.2. Sample Collection

To evaluate the dynamic of AMR rates in the microbiota of broilers throughout the growing period, commensal *E. coli* was selected as sentinel bacterium [20,25].

For this purpose, animals were randomly selected from each experimental group and caeca samples were collected. Three different sampling moments were established: at arrival (day-old chicks), at the mid-period (21 days old) and at the end of the production cycle (42 days of age). On arrival day, animals were selected and sampled just before being delivered to the houses (30 samples). Then, cecal samples were collected per each treatment (60 samples farm condition/house). Caeca were taken individually and placed in sterile jars. Samples were processed within 24 h after collection.

### 2.3. E. coli Isolation

First, cecal content was removed and homogenized. Then, pools of six animals from the same experimental group were prepared: 5 pools from day-old-chicks (30 samples), 10 pools from animals in CFC at mid-period (60 samples), 10 from animals in IFC at mid-period (60 samples), 10 pools from animals in CFC at the end of the growing period (60 samples) and 10 pools from animals in IFC at the end of the growing period (60 samples). The pools’ content was cultured directly onto a Coliform Chromogenic agar (Scharlab, S.L., Barcelona, Spain) in duplicate, and agar plates were incubated at 37 ± 1 °C for 24 h. After incubation, suspected colonies were streaked onto a nutrient medium (Scharlab, S.L., Barcelona, Spain) and incubated at 37 ± 1 °C for 24 h. Then, API-20E test (Biomerieux, S.L., Barcelona, Spain) was performed to confirm *E. coli*.

### 2.4. Antimicrobial Susceptibility Testing

The protocol established to study the antimicrobial susceptibility of the isolates was according to Montoro-Dasi et al. (2020) [20]. Briefly, the bacteria were inoculated onto Mueller-Hinton agar (Scharlab, S.L., Barcelona, Spain) and the antibiotic discs were added. Plates were incubated at 37 ± 1 °C for 24 h. The analysis was carried out according to the European Committee on Antimicrobial Susceptibility Testing guidelines [28] and the source for zone diameters used for interpretation of the test was: http://www.eucast.org/clinical_breakpoints/ (15 September 2020). The AMAs selected were those set forth in Decision 2013/652 [29], including ciprofloxacin (CIP, 5 µg), nalidixic acid (NAL, 30 µg), ampicillin (AMP, 10 µg), cefotaxime (CTX, 30 µg), ceftazidime (CAZ, 30 µg), chloramphenicol (CHL, 5 µg), trimethoprim-sulfamethoxazole (SXT, 1.25/23.75 µg), colistin (CST, 10 µg), azithromycin (AZM, 15 µg), tigecycline (TGC, 15 µg), gentamycin (GEN, 10 µg) and trimethoprim (TMP, 5 µg). MDR was defined as acquired resistance to at least one agent in three or more antimicrobial classes [20,25].

### 2.5. Statistical Analysis

Statistical Analysis was performed according to Montoro-Dasi et al. (2020) [20]. A Generalized Linear Model (GLM) test was used to compare the AMR and MDR rates between farm conditions (CFC vs. IFC) and between sampling moments (arrival day, mid-period and slaughter day). To do so, we fitted GLM where the occurrence of resistance was the response variable and experimental group was the fixed effect. For this analysis, the error was designated as having a binomial distribution and the probit link function was used. Binomial data for each sample were assigned 1 if the *E. coli* isolates were resistant or 0 if not. Similarly, AMR rates of each antibiotic throughout the growing period (arrival day, mid-period and slaughter day) were evaluated, using a GLM as previously. A *p*-value of <0.05 was considered to indicate a statistically significant difference. Analyses were carried out using a commercially available software application (SPSS 24.0 software package, SPSS Inc., Chicago, IL, USA, 2002).

## 3. Results

During this experiment, all the productive parameters, including mortality rates, animals’ weight, feed intake and feed conversion rate (Table 2), were according to the breed standards [30] and no intestinal signs or disease were observed. Thus, no AMAs were administered. In this study, a total of 45 pools of cecal content were analyzed in duplicate, and all of them were culture-positive for *E. coli* (*n* = 90).

### 3.1. Prevalence of Antimicrobial Resistance and Multidrug Resistance

**Figure 1:**
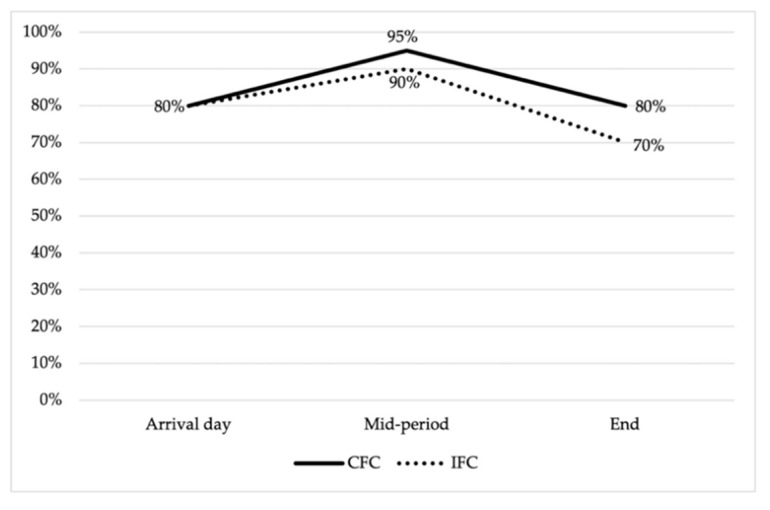
Antimicrobial resistant *E. coli* isolates dynamic for commercial (CFC) and improved farm conditions (IFC) throughout the growing period. No statistically significant differences were observed.

From all *E. coli* isolates, 83.3% (*n* = 75/90) were resistant to at least one of the 12 AMAs tested, and no statistically significant differences were found between replicates. In addition, no statistically significant differences were found between the percentage of resistant *E. coli* strains isolated from the two sampling groups (CFC vs. IFC) (*p*-value > 0.05) (Figure 1).

**Figure 2:**
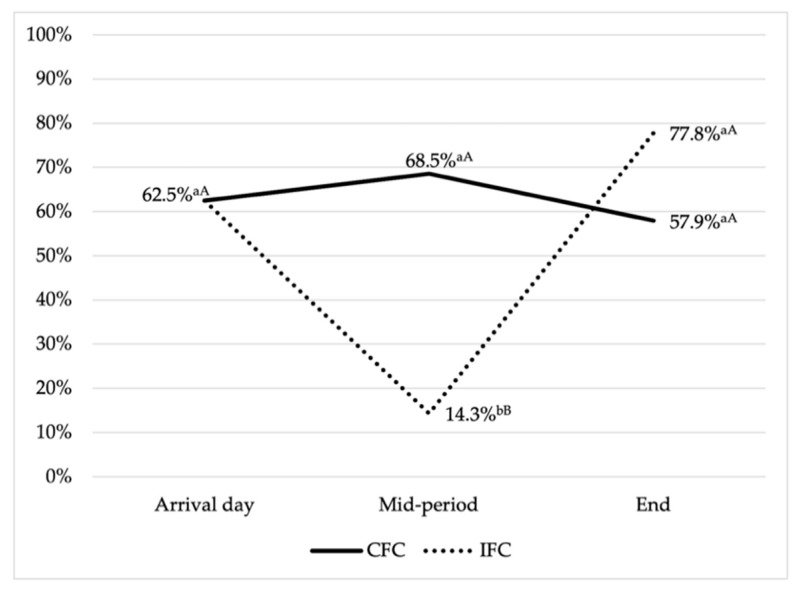
Multidrug-resistant *E. coli* isolates dynamic for commercial (CFC) and improved farm conditions (IFC) throughout the growing period. ^a,b^: Different superscripts means significant differences within group with a *p*-value < 0.05. ^A,B^: Different superscripts indicate significant differences between groups with a *p*-value < 0.05.

Furthermore, 57.3% of the resistant isolates (*n* = 43/75) showed a MDR pattern, with statistically significant differences between experimental groups (Figure 2). At the onset of the growing period, 62.5% of the isolates (*n* = 5/8) were MDR. For CFC, similar rates were maintained until the end of rearing, with a total of 68.8% (*n* = 11/16) and 57.9% (*n* = 11/19) of MDR isolates at mid-period and on slaughter day, respectively. However, for IFC group there were statistically significant differences between sampling moments (*p* < 0.05): mid-period samples (14.3%, *n* = 2/14) displayed a lower level of MDR isolates than those obtained from animals at end of the growing period (77.8%, *n* = 14/18). Moreover, when the percentages of MDR were analyzed between experimental groups, statistically significant differences were found at mid-period (*p* < 0.05).

*E. coli* AMR rates obtained against the different AMAs tested over time for both experimental groups are described in Table 3.

### 3.2. Antimicrobial Resistance Patterns

**Figure 3:**
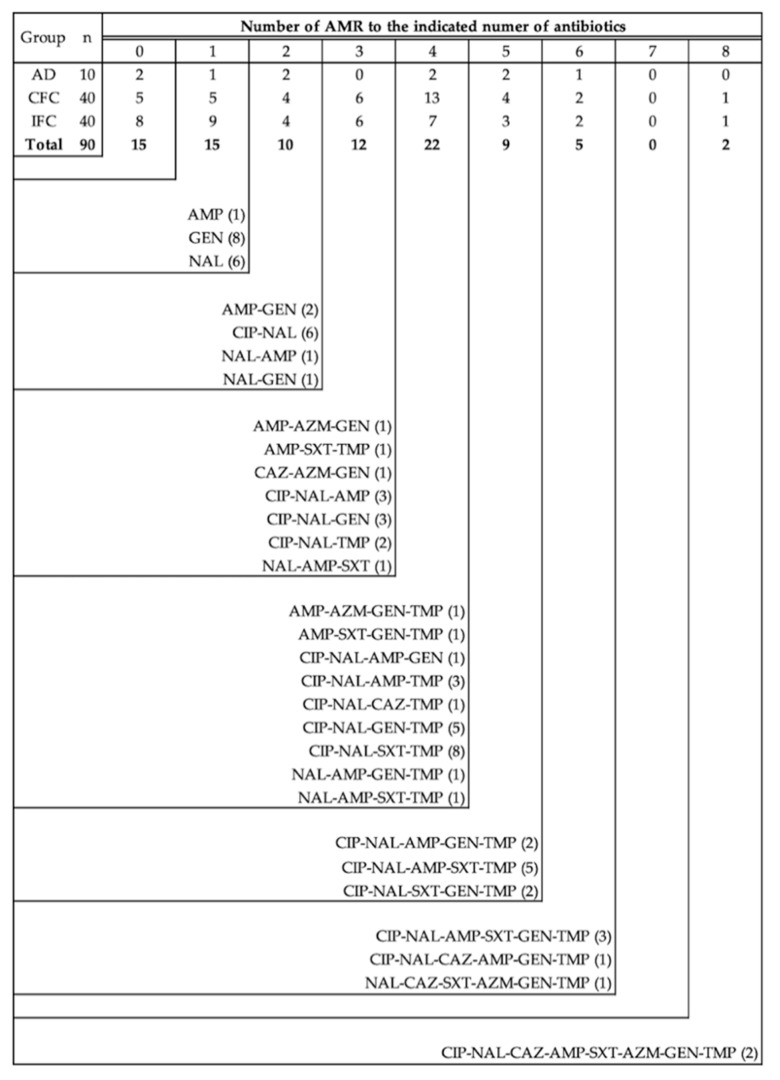
Number of *E. coli* strains isolated resistant to the different number of antimicrobials tested and their antimicrobial resistance pattern, according to commercial (CFC) and improved (IFC) farm conditions. n: total number of isolates from each experimental group. AMR: antimicrobial resistances, AD: arrival day, CFC: Commercial farm conditions, IFC: Improved farm conditions, CIP: ciprofloxacin, NAL: nalidixic acid, AMP: ampicillin, CTX: cefotaxime, CAZ: ceftazidime, CHL: chloramphenicol, SXT: trimethoprim-sulfamethoxazole, CST: colistin, AZM: azithromycin, TGC: tigecycline, GEN: gentamycin, TMP: trimethoprim.

AMR patterns are described in Figure 3. At the arrival day, 20% (*n* = 2) of the isolates were susceptible to all the AMAs tested, 10% (*n* = 1) of the isolates were resistant to only 1 AMA, 20% (*n* = 2) to 2, 20% (*n* = 2) to 4, 20% (*n* = 2) to 5, and 10% (*n* = 1) to 6.

For CFC, 25% (*n* = 5) of the isolates were completely susceptible, 2.5% (*n* = 1) were resistant to one of the 12 AMAs tested, 10% (*n* = 4) to 2, 15% (*n* = 6) to 3, 32.5% (*n* = 13) to 4, 10% (*n* = 4) to 5, 5% (*n* = 2) to 6, and only 2.5% (*n* = 1) were resistant to 8 of the AMAs tested.

Finally, for IFC, 20% (*n* = 8) of the E. coli isolates were susceptible to all the AMAs analysed, 22.5% (*n* = 9) of the isolates were resistant to 1 AMA, 10% (*n* = 4) to 2, 15% (*n* = 6) to 3, 17.5% (*n* = 7) to 4, 7.5% (*n* = 3) to 5, 5% (*n* = 2) to 6, and only 2.5% (*n* = 1) were resistant to 8 of the AMAs tested.

Overall, 34 different resistant patterns were observed, and the most prevalent were GEN (*n* = 8), CIP-NAL-SXT-TMP (*n* = 8), NAL (*n* = 6), CIP-NAL (*n* = 6), CIP-NAL-GEN-TMP (*n* = 5) and CIP-NAL-AMP-SXT-TMP (*n* = 5) (Figure 3).

## 4. Discussion

Despite the fact that no AMAs were administered during the experiment, it was observed that 83.3% of *E. coli* isolates obtained were AMR, and 57.3% of them were MDR, with slight variations between sampling moments. These data are in line with those reported by the last European Food Safety Authority (EFSA) report [11], and could be explained by a vertical or a horizontal resistance acquisition from breeders [31,32] or the environment [20,33], respectively.

At the beginning of the study, on arrival day, the animals presented 80% of resistant *E. coli* isolates, and 62.5% of them were MDR. These results show the importance of AMR and MDR acquired from the breeding, hatching or transport environment [34,35]. It has been reported that day-old chicks could be colonized by direct vertical transmission through breeders’ microbiota [36] or by the resistant bacteria persistent in the hatchery or on delivery transport surfaces [33,37,38], as they are an important threat requiring strict management control in the initial stages to reduce the selective AMR/MDR pressure on breeders, hatcheries and farm environments [9,39].

Among the most relevant results observed in the dynamics of AMAs studied, the highest resistances were observed against ciprofloxacin, nalidixic acid and ampicillin, in line with results reported by the EFSA [11]. It is important to highlight the absence of bacteria resistant to colistin and trimethoprim, as they are critically important AMAs, reserved to treat serious infections caused by MDR bacteria in human medicine [1]. These results reveal that the strategies implemented by governments and poultry industry to control the use of critical AMAs, such as “stop-colistin”, are having an important effect at field level [40]. In line with these findings, further efforts are needed to achieve a greater decrease in the use of other AMAs.

Moreover, in this study at the end of the growing period, resistant bacteria to ceftazidime and azithromycin appeared, and the resistant bacteria to gentamycin and trimethoprim increased. This could be explained by horizontal transmission of resistance genes from the environment, which is considered a critical point in livestock production. Several authors demonstrated that horizontal transmission of resistances from the environment could be more important than vertical transmission in broiler production [33]. In fact, previous studies demonstrated that residual feces or dust are important reservoirs for resistant bacteria and AMR genes between different flocks in commercial farms due to the high survival of resistant microorganisms after cleaning and disinfection procedures [41,42,43,44], with the application of proper cleaning and disinfection protocols being mandatory to avoid the survival of bacteria [45,46].

In this regard, it is demonstrated that an increase in animal welfare promotes the presence of beneficial microbiota and the integrity of the intestinal epithelium. As a consequence, the protective mechanisms are working perfectly and the interactions between environmental and intestinal bacteria are reduced. In contrast, stress situations such as the arrival to new facilities or the high-density levels presented at the end of the growing period reduce the effectiveness of these protective mechanisms, increasing the colonization of potential pathogens and resistant bacteria to the intestinal tract of broilers, increasing interactions and transmission of resistant genes [47,48,49,50]. In this sense, animal welfare could be considered as preventive medicine, promoting immunologically stronger animals that are better able to cope with infectious diseases without administration of AMAs [15,48,51]. However, in this study it was observed that although animals subjected to less intensive production conditions showed a lower level of MDR at mid-period, at the end of the growing stage the presence of AMR and MDR was particularly high, regardless of the poultry being under less or more intensive conditions, at around 70% and 77.8%, respectively. This fact could be explained by the high AMR rates on the arrival day, and the short time of rearing (42 days), highlighting the importance of controlling the use of AMAs in the first stages of the poultry production system [39]. In addition, it is important not to forget that at the end of the growing period, when the highest levels of AMR have been observed, animals are handled for transport to the slaughterhouse, which could involve an increase in stress, intestinal dysbiosis and excretion of microorganisms in feces just before processing of the carcasses, constituting an important threat to consumers [52,53,54,55]. Therefore, it is essential to develop more accurate and cost-effective techniques to be applied at farm level to avoid the presence of AMR and MDR microorganisms upon arrival at the slaughterhouse.

## 5. Conclusions

In conclusion, AMR and MDR are present throughout the growing period, although no AMAs were administered. Moreover, although a lower level of MDR was observed at mid-period in animals reared under less intensive farming conditions, no differences were found between the two experimental groups at the end of the growing period. Further studies are needed to evaluate how management could reduce the presence of AMR and MDR bacteria in poultry production at all production stages.

## Figures and Tables

**Table 1:** Composition of starter and grower diets.

Analytical Constituents	Diet	
	Starter (d 1–21) (%)	Grower (d 22–42) (%)
Crude Fat	3.5	3.1
Crude Protein	20.5	19.4
Crude Fibre	2.6	3.1
Crude Ash	6.6	5.0
Lysine	1.14	1.13
Methionine	0.62	0.51
Calcium	1.00	0.78
Phosphorus Available	0.69	0.51
Sodium	0.15	0.14
Ingredients	Corn, soy flour, wheat, soy oil, calcium carbonate, monocalcium phosphate, sodium chloride	Corn, soy flour, rice bran, calcium carbonate, sodium chloride

**Table 2:** Mortality rate (MR), body weight (BW), feed intake (FI) and feed conversion rate (FCR) of the animals for both experimental groups—commercial farm conditions (CFC) and improved farm conditions (IFC)—throughout the growing period.

Days of Life	CFC				IFC			
	MR (%)	BW (g)	FI (Kg)	FCR	MR (%)	BW (g)	FI (Kg)	FCR
7	1.47	157.73	0.13	1.19	0.98	160.42	0.13	1.12
14	0.50	413.17	0.37	1.35	1.49	428.59	0.36	1.38
21	0	788.25	0.71	2.02	0	789.15	0.67	1.89
28	0	1234.59	1.17	2.66	0	1233.51	1.09	2.46
35	0	1810.06	1.45	2.54	0	1788.30	1.27	2.27
42	0	2471.14	1.51	2.25	0	2461.13	1.36	2.09

**Table 3:** Antimicrobial resistance rates obtained for each antibiotic in different sampling moments and experimental groups (commercial vs. improved farm conditions) throughout the growing period.

Experimental Group	Sampling Moment	*n*	CIP	NAL	CTX	CAZ	AMP	CHL	SXT	CST	AZM	TGC	GEN	TMP
CFC	Arrival day	10	70 ^a^	70	0	0	50	0	30 ^ab^	0	0 ^b^	0	30 ^bc^	40 ^a^
	Mid-period	20	60 ^a^	75	0	0	30	0	55 ^a^	0	0 ^b^	0	10 ^c^	55 ^a^
	End	20	60 ^a^	65	0	2	45	0	10 ^b^	0	1 ^ab^	0	75 ^a^	60 ^a^
IFC	Arrival day	10	70 ^a^	70	0	0	50	0	30 ^ab^	0	0 ^b^	0	30 ^bc^	40 ^a^
	Mid-period	20	30 ^b^	55	0	0	20	0	10 ^b^	0	0 ^b^	0	15 ^c^	10 ^b^
	End	20	50 ^a^	65	0	4	35	0	35 ^a^	0	5 ^a^	0	65 ^ab^	55 ^a^

^a–c^: Different superscripts in each antibiotic means significant differences with a *p*-value < 0.05. *n*: total of isolates from each experimental group in each sampling moment. CFC: Commercial farm conditions, IFC: Improved farm conditions, CIP: ciprofloxacin, NAL: nalidixic acid, AMP: ampicillin, CTX: cefotaxime, CAZ: ceftazidime, CHL: chloramphenicol, SXT: trimethoprim-sulfamethoxazole, CST: colistin, AZM: azithromycin, TGC: tigecycline, GEN: gentamycin, TMP: trimethoprim.

## Data Availability

The data presented in this study are available in Montoro-Dasi, L.; Villagra, A.; Sevilla-Navarro, S.; Pérez-Gracia, M.T.; Vega, S.; Marin, C. Commensal Escherichia coli Antimicrobial Resistance and Multidrug-Resistance Dynamics during Broiler Growing Period: Commercial vs. Improved Farm Conditions. *Animals* 2021, accepted manuscript.

## References

[1] WHO (World Health Organization) (2019). Critically Important Antimicrobials for Human Medicine.

[2] Marshall B.M., Levy S.B. (2011). Food Animals and Antimicrobials: Impacts on Human Health. Clin. Microbiol. Rev..

[3] Chang Q., Wang W., Regev-Yochay G., Lipsitch M., Hanage W.P. (2015). Antibiotics in agriculture and the risk to human health: How worried should we be?. Evol. Appl..

[4] Founou L.L., Founou R.C., Essack S.Y. (2016). Antibiotic Resistance in the Food Chain: A Developing Country-Perspective. Front. Microbiol..

[5] Horigan V., Kosmider R.D., Horton R.A., Randall L., Simons R.R.L. (2016). An assessment of evidence data gaps in the investigation of possible transmission routes of extended spectrum β-lactamase producing *Escherichia coli* from livestock to humans in the UK. Prev. Vet. Med..

[6] Liu Y.Y., Wang Y., Walsh T.R., Yi L.X., Zhang R., Spencer J., Doi Y., Tian G., Dong B., Huang X. (2016). Emergence of plasmid-mediated colistin resistance mechanism MCR-1 in animals and human beings in China: A microbiological and molecular biological study. Lancet Infect. Dis..

[7] Sharma C., Rokana N., Chandra M., Singh B.P., Gulhane R.D., Gill J.P.S., Ray P., Puniya A.K., Panwar H. (2018). Antimicrobial resistance: Its surveillance, impact, and alternative management strategies in dairy animals. Front. Vet. Sci..

[8] ESVAC (European Medicines Agency) European Database of Sales of Veterinary Antimicrobial Agents. https://esvacbi.ema.europa.eu/analytics/saw.dll?PortalPages.

[9] Aarestrup F.M. (2015). The livestock reservoir for antimicrobial resistance: A personal view on changing patterns of risks, effects of interventions and the way forward. Philos. Trans. R. Soc. B Biol. Sci..

[10] Khurana A., Sinha R., Nagaraju M. (2017). Antibiotic Resistance in Poultry Environment. Spread of Resistance from Poultry Farm to Agricultural Field. Cent. Sci. Environ..

[11] EFSA and ECDC (European Food Safety Authority and European Centre for Disease Prevention and Control) (2020). The European Union Summary Report on Antimicrobial Resistance in zoonotic and indicator bacteria from humans, animals and food in 2017/2018. EFSA J..

[12] Apostolakos I., Piccirillo A. (2018). A review on the current situation and challenges of colistin resitance in poultry production. Avian Pathol..

[13] EMA (European Medicines Agency) (2020). Sales of Veterinary Antimicrobial Agents in 31 European Countries in 2018 (EMA/24309/2020). Trends from 2010 to 2018 Tenth ESVAC Report.

[14] PRAN (Plan Nacional de Resistencia Antibióticos) (2020). Informe Anual PRAN Junio 2019—Junio. https://resistenciaantibioticos.es/es/publicaciones/informe-anual-2019-2020-plan-nacional-frente-la-resistencia-los-antibioticos.

[15] Rojo-Gimeno C., Postma M., Dewulf J., Hogeveen H., Lauwers L., Wauters E. (2016). Farm-economic analysis of reducing antimicrobial use whilst adopting improved management strategies on farrow-to-finish pig farms. Prev. Vet. Med..

[16] Gomes A.V.S., Quinteiro-Filho W.M., Ribeiro A., Ferraz-de-Paula V., Pinheiro M.L., Baskeville E., Akamine A.T., Astolfi-Ferreira C.S., Ferreira A.J.P., Palermo-Neto J. (2014). Overcrowding stress decreases macrophage activity and increases *Salmonella* Enteritidis invasion in broiler chickens. Avian Pathol..

[17] Rouger A., Tresse O., Zagorec M. (2017). Bacterial Contaminants of Poultry Meat: Sources, Species, and Dynamics. Microorganisms.

[18] Swaggerty C.L., Callaway T.R., Kogut M.H., Piva A., Grilli E. (2019). Modulation of the immune response to improve health and reduce foodborne pathogens in poultry. Microorganisms.

[19] Soleimani A.F., Zulkifli I., Hair-Bejo M., Ebrahimi M., Jazayeri S.D., Hashemi S.R., Meimandipour A., Goh Y.M. (2012). Epigenetic modification: Possible approach to reduce *Salmonella enterica* serovar enteritidis susceptibility under stress conditions. Avian Pathol..

[20] Montoro-Dasi L., Villagra A., Sevilla-Navarro S., Pérez-Gracia M.T., Vega S., Marin C. (2020). The dynamic of antibiotic resistance in commensal *Escherichia coli* throughout the growing period in broiler chickens: Fast-growing vs. slow-growing breeds. Poult. Sci..

[21] Gocsik É., Brooshooft S.D., de Jong I.C., Saatkamp H.W. (2016). Cost-efficiency of animal welfare in broiler production systems: A pilot study using the Welfare Quality^®^ assessment protocol. Agric. Syst..

[22] El-Deek A., El-Sabrout K. (2019). Behaviour and meat quality of chicken under different housing systems. World Poult. Sci. J..

[23] Sirri F., Castellini C., Bianchi M., Petracci M., Meluzzi A., Franchini A. (2011). Effect of fast-, medium- and slow-growing strains on meat quality of chickens reared under the organic farming method. Animal.

[24] Lusk J.L. (2018). Consumer preferences for and beliefs about slow growth chicken. Poult. Sci..

[25] EFSA and ECDC (European Food Safety Authority and European Centre for Disease Prevention and Control) (2019). Technical specifications on harmonised monitoring of antimicrobial resistance in zoonotic and indicator bacteria from food-producing animals and food. EFSA J..

[26] RD (Royal Degree) (2013). Spain. Royal Degree 53/2013, 1st of February, Por el Que se Establecen las Normas Básicas Aplicables Para la Protección de los Animales Utilizados en Experimentación y Otros Fines Científicos, Incluyendo la Docencia. https://www.boe.es/diario_boe/txt.php?id=BOE-A-2013-1337.

[27] Kersia Group Pig & Poultry Farming. https://www.kersia-group.com/activities/pig-poultry-farming/.

[28] Matuschek E., Brown D.F.J., Kahlmeter G. (2014). Development of the EUCAST disk diffusion antimicrobial susceptibility testing method and its implementation in routine microbiology laboratories. Clin. Microbiol. Infect..

[29] EC (European Commission) (2013). Commission Implementing Decission, of 12 November 2013, on the Monitoring and Reporting of Antimicrobial Resistance in Zoonotic and Commensal Bacteria (2013/652/EU). https://eur-lex.europa.eu/legal-content/EN/TXT/?uri=CELEX%3A32013D0652.

[30] (2019). Aviagen Ross 308: Broiler Performance Objectives.

[31] Osman K.M., Kappell A.D., Elhadidy M., Elmougy F., El-Ghany W.A.A., Orabi A., Mubarak A.S., Dawoud T.M., Hemeg H.A., Moussa I.M.I. (2018). Poultry hatcheries as potential reservoirs for antimicrobial-resistant *Escherichia coli*: A risk to public health and food safety. Sci. Rep..

[32] Marin C., Sevilla-Navarro S., Lonjedo R., Catalá-Gregori P., Ferrús M.A., Vega S., Jiménez-Belenguer A. (2020). Genotyping and molecular characterization of antimicrobial resistance in thermophilic *Campylobacter* isolated from poultry breeders and their progeny in Eastern Spain. Poult. Sci..

[33] Oikarainen P.E., Pohjola L.K., Pietola E.S., Heikinheimo A. (2019). Direct vertical transmission of ESBL/pAmpC-producing *Escherichia coli* limited in poultry production pyramid. Vet. Microbiol..

[34] Poulsen L.L., Thøfner I., Bisgaard M., Christensen J.P., Olsen R.H., Christensen H. (2017). Longitudinal study of transmission of *Escherichia coli* from broiler breeders to broilers. Vet. Microbiol..

[35] Dame-Korevaar A., Fischer E.A.J., van der Goot J., Velkers F., van den Broek J., Veldman K., Ceccarelli D., Mevius D., Stegeman A. (2019). Effect of challenge dose of plasmid-mediated extended-spectrum β-lactamase and AmpC β-lactamase producing *Escherichia coli* on time-until-colonization and level of excretion in young broilers. Vet. Microbiol..

[36] Nilsson O., Börjesson S., Landén A., Bengtsson B. (2014). Vertical transmission of *Escherichia coli* carrying plasmid-mediated AmpC (pAmpC) through the broiler production pyramid. J. Antimicrob. Chemother..

[37] Projahn M., Daehre K., Roesler U., Friese A. (2017). Extended-spectrum-beta-lactamaseand plasmid-encoded cephamycinaseproducing enterobacteria in the broiler hatchery as a potential mode of pseudovertical transmission. Appl. Environ. Microbiol..

[38] Projahn M., Daehre K., Semmler T., Guenther S., Roesler U., Friese A. (2018). Environmental adaptation and vertical dissemination of ESBL-/pAmpC-producing *Escherichia coli* in an integrated broiler production chain in the absence of an antibiotic treatment. Microb. Biotechnol..

[39] Dierikx C.M., van der Goot J.A., Smith H.E., Kant A., Mevius D.J. (2013). Presence of ESBL/AmpC -Producing *Escherichia coli* in the Broiler Production Pyramid: A Descriptive Study. PLoS ONE.

[40] WHO (World Health Organization) European Strategic Action Plan on Antibiotic Resistance 2011. http://www.euro.who.int/__data/assets/pdf_file/0008/147734/wd14E_AntibioticResistance_111380.pdf.

[41] Davies R., Wales A. (2019). Antimicrobial Resistance on Farms: A Review Including Biosecurity and the Potential Role of Disinfectants in Resistance Selection. Compr. Rev. Food Sci. Food Saf..

[42] Marin C., Balasch S., Vega S., Lainez M. (2011). Sources of *Salmonella* contamination during broiler production in Eastern Spain. Prev. Vet. Med..

[43] Chuppava B., Keller B., Abd El-Wahab A., Sürie C., Visscher C. (2019). Resistance Reservoirs and Multi-Drug Resistance of Commensal *Escherichia coli* from Excreta and Manure Isolated in Broiler Houses with Different Flooring Designs. Front. Microbiol..

[44] Luiken R.E.C., Van Gompel L., Bossers A., Munk P., Joosten P., Hansen R.B., Knudsen B.E., García-Cobos S., Dewulf J., Aarestrup F.M. (2020). Farm dust resistomes and bacterial microbiomes in European poultry and pig farms. Environ. Int..

[45] Carrique-Mas J.J., Marín C., Breslin M., McLaren I., Davies R. (2009). A comparison of the efficacy of cleaning and disinfection methods in eliminating *Salmonella* spp. from commercial egg laying houses. Avian Pathol..

[46] Maertens H., De Reu K., Meyer E., Van Coillie E., Dewulf J. (2019). Limited association between disinfectant use and either antibiotic or disinfectant susceptibility of *Escherichia coli* in both poultry and pig husbandry. BMC Vet. Res..

[47] Burkholder K.M., Thompson K.L., Einstein M.E., Applegate T.J., Patterson J.A. (2008). Influence of stressors on normal intestinal microbiota, intestinal morphology, and susceptibility to *Salmonella* Enteritidis colonization in broilers. Poult. Sci..

[48] Dawkins M.S. (2019). Animal welfare as preventative medicine. Anim. Welf..

[49] He J., He Y., Pan D., Cao J., Sun Y., Zeng X. (2019). Associations of gut microbiota with heat stress-induced changes of growth, fat deposition, intestinal morphology, and antioxidant capacity in ducks. Front. Microbiol..

[50] Mandal R.K., Jiang T., Wideman R.F., Lohrmann T., Kwon Y.M. (2020). Microbiota Analysis of Chickens Raised Under Stressed Conditions. Front. Vet. Sci..

[51] Burbarelli M.F.C., Merseguel C.E.B., Ribeiro P.A.P., Lelis K.D., Polycarpo G.V., Carão A.C.P., Bordin R.A., Fernandes A.M., Souza R.L.M., Moro M.E.G. (2015). The effects of two different cleaning and disinfection programs on broiler performance and microbiological status of broiler houses. Rev. Bras. Cienc. Avic..

[52] Marin C., Lainez M. (2009). *Salmonella* detection in feces during broiler rearing and after live transport to the slaughterhouse. Poult. Sci..

[53] Sevilla-Navarro S., Marin C., Cortés V., García C., Catalá-Gregori P. (2020). *Campylobacter* prevalence and risk factors associated with exceeding allowable limits in poultry slaughterhouses in Spain. Vet. Rec..

[54] Althaus D., Zweifel C., Stephan R. (2017). Analysis of a poultry slaughter process: Influence of process stages on the microbiological contamination of broiler carcasses. Ital. J. Food Saf..

[55] Gregova G., Kmetova M., Kmet V., Venglovsky J., Feher A. (2012). Antibiotic resistance of *Escherichia coli* isolated from a poultry slaughterhouse. Ann. Agric. Environ. Med..

